# VEXAS in a patient with hypereosinophilia and Sweet’s-like lesions

**DOI:** 10.1016/j.jdcr.2024.08.024

**Published:** 2024-09-07

**Authors:** Martha J. Longley, Rebecca G. Gaffney, Jeffrey S. Smith, Mia S. DeSimone, Michael E. Weinblatt, Joseph F. Merola

**Affiliations:** a Harvard Medical School, Boston, Massachusetts; b Department of Dermatology, Harvard Medical School, Brigham and Women’s Hospital, Boston, Massachusetts; c Department of Pathology, Harvard Medical School, Brigham and Women's Hospital, Boston, Massachusetts; d Division of Rheumatology, Inflammation and Immunity, Brigham and Women’s Hospital, Boston, Massachusetts; e Division of Rheumatology, Department of Dermatology and Department of Medicine, UT Southwestern Medical Center, Dallas, Texas

**Keywords:** autoinflammatory, hypereosinophilia, VEXAS

## Introduction

VEXAS (Vacuoles, E1 enzyme, X-linked, Autoinflammatory, Somatic) syndrome is a recently described disorder of myeloid progenitor cells that presents as a late-onset, progressive autoimmune disease, often with skin manifestations, that predominantly affects older males. It is caused by acquired mutations in *UBA1* on the X-chromosome.^1^ *UBA1* encodes ubiquitin-activating enzyme 1 (UBA1), a regulator of ubiquitination—a post-translational modification that in certain contexts modulates protein function and in others proteasomal degradation.^2^ Initial reports of patients with VEXAS described somatic mutations at methionine 41 that produce a catalytically inactive UBA1, though other mutations also cause the disease.^1^ Pathogenic mutations occur in hematopoietic stem cells and have been shown to affect mature myeloid cells including monocytes, neutrophils, megakaryocytes, and erythroblasts. Inactive UBA1 disrupts normal protein turnover, which triggers cell stress and uncontrolled inflammation. Phenotypes are still being described and often include dermatological, pulmonary, vascular, and cartilaginous inflammation. Hematologic findings of VEXAS often include macrocytic anemia, macrocytosis, and the presence of vacuoles in myeloid and erythroid precursor cells, and the disease can be associated with venous thromboembolism, plasma cell dyscrasia, and myelodysplastic syndrome.^3^ Cutaneous symptoms appear to affect ∼90% of patients^4^ and include relapsing polychondritis, periorbital edema, polyarteritis nodosa, erythema nodosum, livedo reticularis, and Sweet’s-like erythematous nodules.^4^ The most commonly reported skin histopathologic findings in VEXAS include neutrophilic dermatosis, especially the histiocytoid variant, leukocytoclastic vasculitis, and perivascular dermatitis.^5^^,^^6^ Here we highlight the phenotypic variation in VEXAS by discussing both the cutaneous and histological manifestations in a confirmed VEXAS patient who presented with hypereosinophilia, Sweet’s-like lesions, and arthralgias.

## Case report

**Fig 1:**
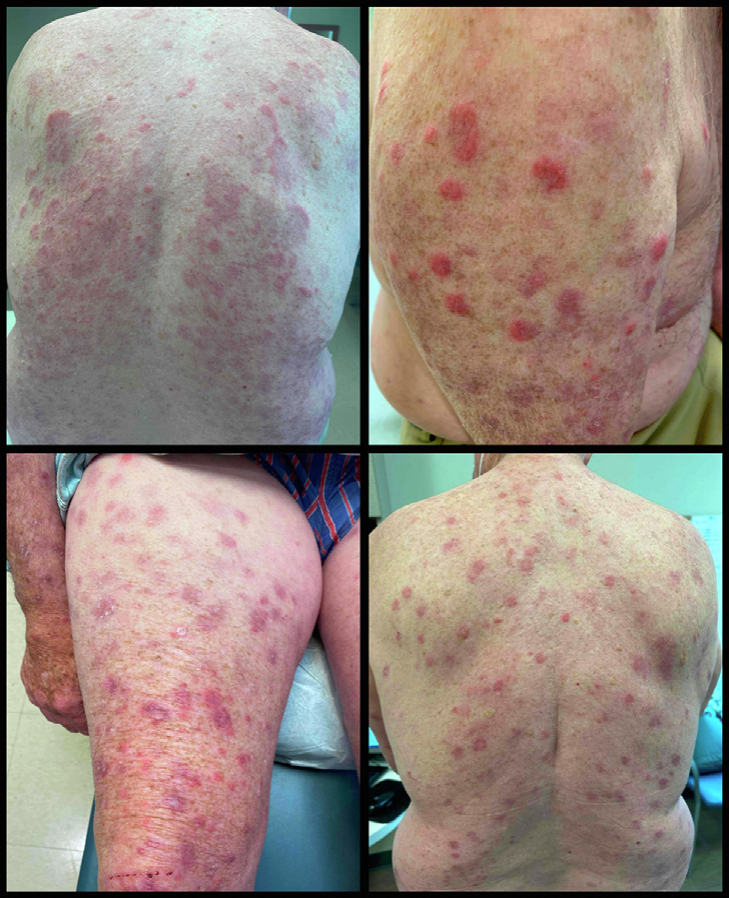
Representative images of Sweet’s-like papules.

**Fig 2:**
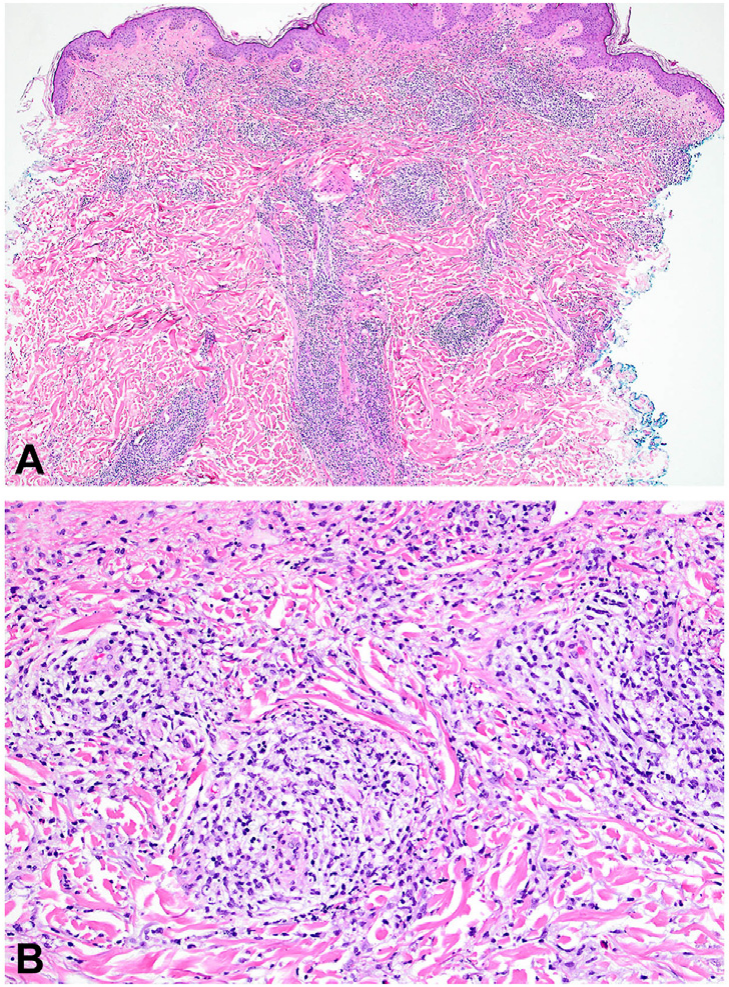
Histopathology of skin punch biopsy from left chest. **A,** At low power, there is marked superficial and deep perivascular, perifollicular, and interstitial inflammatory infiltrate. **B,** At higher power, the inflammatory infiltrate is composed of lymphocytes, histiocytes, neutrophils, and scattered eosinophils.

A 76-year-old man presented to our dermatology clinic with diffuse, edematous, red-to-violaceous papules and plaques that began 8 months prior (Fig 1). Skin biopsies revealed minimal spongiosis with a superficial to deep dermal perivascular, periadnexal, and interstitial lymphohistiocytic infiltrate with admixed neutrophils and eosinophils (Fig 2). Although the clinical differential diagnosis favored Sweet syndrome, a hypersensitivity reaction was favored based on the histopathologic findings. Extensive neoplastic workup was unremarkable. Laboratory studies were notable for peripheral eosinophilia (24%; NL: <5%). His hydrochlorothiazide and simvastatin were discontinued out of concern for a drug-induced inflammatory reaction. He was started on a course of prednisone (60 mg/day).

**Fig 3:**
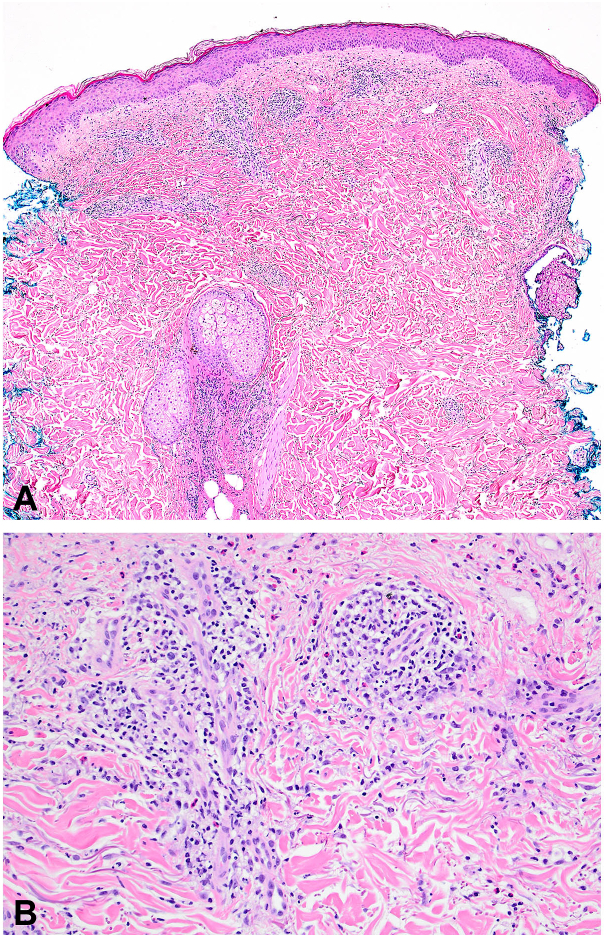
Histopathology of skin punch biopsy from left upper back. **A,** At low power, there is superficial and mid-dermal perivascular, perifollicular, and interstitial inflammatory infiltrate. **B,** At higher power, the inflammatory infiltrate is composed of lymphocytes, histiocytes, neutrophils, and numerous eosinophils.

**Fig 4:**
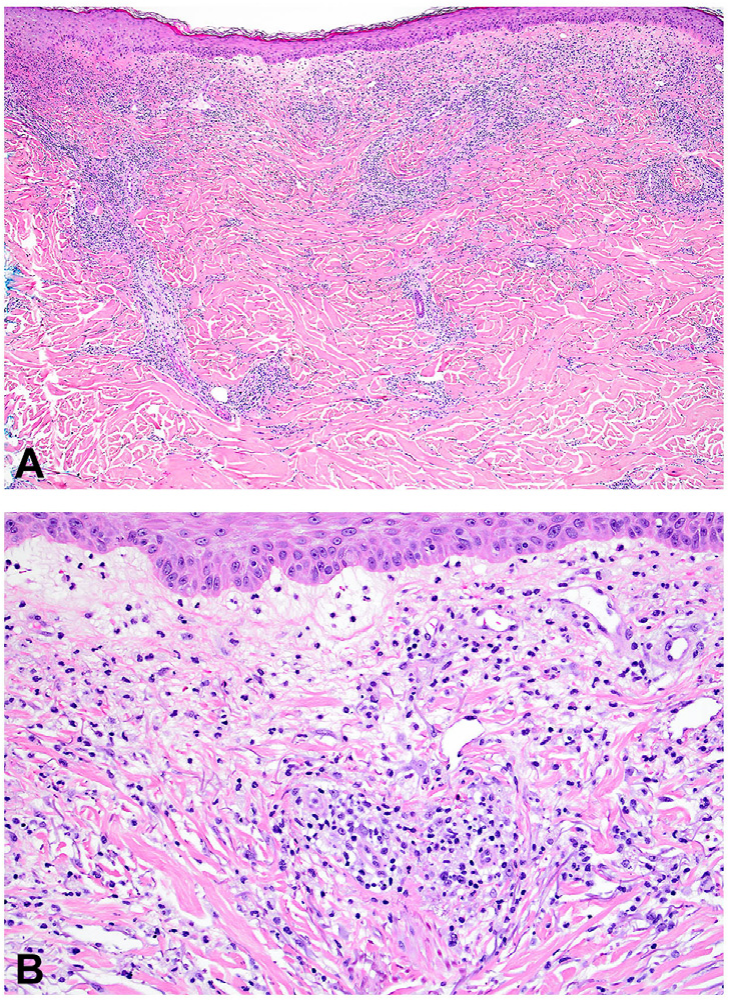
Histopathology of skin punch biopsy from right upper back. **A,** At low power, there is focal papillary dermal edema, superficial, and mid-dermal perivascular, perifollicular and interstitial inflammatory infiltrate. **B,** At higher power, the inflammatory infiltrate is composed of lymphocytes, histiocytes, scattered eosinophils, and numerous neutrophils.

Further along in his clinical course, he began experiencing periodic night sweats and new-onset inflammatory arthritis which impacted his ability to function. Peripheral eosinophilia peaked at 32% (NL <8%, Abs = 1.88, NL <0.9) and C-reactive protein was elevated at 57 mg/L (NL: 1-8 mg/L). Tryptase levels were normal. Peripheral flow cytometry showed a B-cell count of 45 cells/uL (NL >500 cells/uL), consistent with a low-count monoclonal B-cell lymphocytosis. Two punch biopsies of the lesions were taken, which showed mild spongiosis with superficial to mid-dermal perivascular, periadnexal, and interstitial lymphohistiocytic infiltrate with frequent eosinophils and neutrophils, most consistent with a hypersensitivity reaction with an interstitial granuloma annulare-like background (Fig 3). Direct immunofluorescence studies showed no immunoreactivity to IgG, IgA, IgM, C3, or fibrinogen. A positron emission tomography scan 1 month later showed mildly avid mediastinal and hilar nodes consistent with a reactive or inflammatory process. He was started hydroxychloroquine (400 mg/day), methotrexate (escalated to 25 mg/week), and intermittent prednisone courses (up to 60 mg/day) which improved his joint pain. Four months later, the patient presented with persistent juicy plaques covering his body. Punch biopsy from the right upper back revealed mild spongiosis, focal papillary dermal edema, and a superficial to mid-dermal perivascular, periadnexal, and interstitial mixed lymphohistiocytic infiltrate with admixed neutrophils and eosinophils (Fig 4). Although the histologic findings were not entirely specific, a palisaded neutrophilic granulomatous dermatitis was raised in the differential diagnosis. He was started on dapsone (25 mg/day) and leucovorin (10 mg/week) and continued on methotrexate (25 mg/week).

Six months after his initial presentation at dermatology, he continued to have a diffuse, edematous rash with plaques and studded pustules on his trunk and extremities with similar biopsy findings. Due to his persistent rash with atypical histopathology in the setting of inflammatory arthritis, a diagnosis of VEXAS was suspected. At this point, genetic testing revealed a mutation in *UBA1* c.121A > C, p.(Met41Leu) confirming a diagnosis of VEXAS. The patient is currently managed on methotrexate (17.5 mg weekly), leucovorin (40 mg weekly), dapsone (100 mg daily), and prednisone (5 mg daily) with significant improvement in his joint pain and improvement of his cutaneous manifestations. Despite symptomatic disease control, he continues to have mild leukopenia, anemia, and peripheral eosinophilia.

## Discussion

VEXAS is a novel autoinflammatory syndrome with numerous phenotypes that remain incompletely reported. Here we describe a patient who presented with Sweet’s-like clinical lesions with relatively nonspecific histopathologic findings, including mild spongiosis, papillary dermal edema, and superficial to deep perivascular, periadnexal, and interstitial mixed inflammatory infiltrate with varying degrees of neutrophils and eosinophils, inflammatory arthritis, and persistent hypereosinophilia. Current VEXAS treatment approaches center around controlling inflammatory symptoms, with successful pharmacological options reported including corticosteroids, Janus kinase inhibitors, or interleukin-6 inhibition. In severe cases, allogeneic hematopoietic stem cell transplant has been curative for some patients but has also led to death in several instances.^7^

This case is notable because hypereosinophilia is not considered a typical finding of VEXAS and may lead to a delay in genetic testing and diagnosis in an otherwise typical presentation of the disease.^4^^,^^7^, ^8^, ^9^ In a study at the National Institutes of Health, it was also found that of the rarely reported VEXAS patients with hypereosinophilia, all had cutaneous manifestations of the disease such as in our patient.^10^ Thus, dermatologists should be aware of hypereosinophilia and the associated clinical phenotype in the presentation of VEXAS syndrome.

## Conflicts of interest

Dr Merola is a consultant and/or investigator for Amgen, AstraZeneca, Boehringer Ingelheim, Bristol Myers Squibb, Abbvie, Dermavant, Eli Lilly, Incyte, Moonlake, Novartis, Janssen, UCB, Sanofi-Regeneron, Sun Pharma, Biogen, Pfizer, and Leo Pharma. Dr Smith is a consultant and/or investigator for Biogen. Dr Weinblatt receives research funding from Bristol Myers Squibb, Aqtual, Abbvie, and Janssen. He is a consultant for Abbvie, Aclaris, Amgen, Aqtual, Bristol Myers Squibb, GlaxoSmithKline, Lilly, Novartis, Pfizer, Prometheus, and Rani; and also holds stock in Canfite, Immedix, and Scipher. M. Longley and Drs Gaffney and DeSimone have no conflicts of interest to declare. This work has not been presented previously.
